# OCT4 Represses Inflammation and Cell Injury During Orchitis by Regulating CIP2A Expression

**DOI:** 10.3389/fcell.2021.683209

**Published:** 2021-08-26

**Authors:** Ruifeng Zeng, Chengli Jin, Chuchu Zheng, Shaoqi Li, Siyue Qian, Jingsa Pan, Lvhe Wang, Junfeng Zhao, Le Qin

**Affiliations:** ^1^Department of Anesthesiology, The Second Affiliated Hospital and Yuying Children’s Hospital of Wenzhou Medical University, Wenzhou, China; ^2^Department of First Clinical Medical School, Wenzhou Medical University, Wenzhou, China; ^3^Department of Second Clinical Medical School, Wenzhou Medical University, Wenzhou, China; ^4^Department of Pediatric Surgery, Ningbo Women and Children’s Hospital, Wenzhou, China; ^5^Department of Pediatric Surgery, The Second Affiliated Hospital and Yuying Children’s Hospital of Wenzhou Medical University, Wenzhou, China

**Keywords:** orchitis, inflammation, apoptosis, redox equilibrium, OCT4, CIP2A

## Abstract

Octamer-binding transcription factor 4 (OCT4) and cancerous inhibitor of protein phosphatase 2A (CIP2A) are upregulated in testicular cancer and cell lines. However, its contribution to orchitis (testicular inflammation) is unclear and was thus, investigated herein. Cell-based experiments on a lipopolysaccharide (LPS)-induced orchitis mouse model revealed robust inflammation, apoptotic cell death, and redox disorder in the Leydig (interstitial), Sertoli (supporting), and, germ cells. Meanwhile, real-time quantitative PCR revealed low OCT4 and CIP2A levels in testicular tissue and LPS-stimulated cells. A gain-of-function study showed that OCT4 overexpression not only increased CIP2A expression but also repressed LPS-induced inflammation, apoptosis, and redox disorder in the aforementioned cells. Furthermore, the re-inhibition of CIP2A expression by TD-19 in OCT4-overexpressing cells counteracted the effects of OCT4 overexpression on inflammation, apoptosis, and redox equilibrium. In addition, our results indicated that the Keap1-Nrf2-HO-1 signaling pathway was mediated by OCT4 and CIP2A. These findings provide insights into the potential mechanism underlying OCT4- and CIP2A-mediated testicular inflammation.

## Introduction

In the process of mammalian sperm formation, many new antigens, including sperm agglutination antigen-1 (SAGA-1) and hyaluronidase (PH-20), are generated during reproductive cell development after the immune capacity of the organism is established (A et al., 2019). Under physiological conditions, such antigens usually do not trigger harmful immunological reactions in the testes owing to testicular immune privilege. However, infection, trauma, inflammation, and other pathological states may inhibit the immunosuppressive mechanism and result in chronic experimental autoimmune orchitis, which can cause male sterility (Schuppe et al., 2008). Therefore, knowledge about the potential mechanism underlying orchitis progression would be helpful in identifying strategies for the prevention and treatment of this inflammatory condition of the testes.

Octamer-binding transcription factor 4 (OCT4, also known as POU5F1) is a transcription factor in embryonic stem cells and germ cells that has been found in pluripotent primary testicular germ cell tumors, seminoma, and embryonal carcinoma (Jones et al., 2004). It aids in maintaining and regulating pluripotency and is involved in normal cell development by regulating the expression of multiple downstream target genes, such as UTF1 and REX1/ZFP42 (Saijoh et al., 1996). Abnormal OCT4 levels in mice may lead to the abnormal expression of key developmental genes (Boiani et al., 2002); OCT4 also plays a role in regulating human cell differentiation, as observed in human embryos (Lamb and Rizzino, 1998; Tomioka et al., 2002). There are many types of testicular germ cell tumors in adults, each showing differences in cell differentiation (Grigor, 1993; Ohr, 2000). *In vitro* studies of specific cancer types, including seminoma and embryonal carcinoma, have indicated that OCT4 regulates the expression of the oncogenic 1.5-kb alternative PDGF receptor (Kraft et al., 1996; Palumbo et al., 2002). An increasing number of studies are also identifying an anti-inflammatory effect of OCT4 in many types of diseases, including ulcerative colitis-associated colorectal cancer (Yasuda et al., 2011) and LPS-induced acute uterine injury (Xiao et al., 2017), and cells, such as human adipose tissue-derived mesenchymal stem cells (Li et al., 2017). Although the involvement of OCT4 in testicular tumors and inflammation has been elucidated, further investigation its role in testicular inflammation, namely, orchitis, is warranted.

Cancerous inhibitor of protein phosphatase 2A (CIP2A), which inhibits protein phosphatase 2A (PP2A) activity, promotes malignant cell transformation and tumor growth by inhibiting the tumor suppressor activity of PP2A (Junttila et al., 2007; Khanna et al., 2013). As shown in previous studies, CIP2A is overexpressed in several cancer types and is associated with reduced survival in some conditions, such as serous ovarian cancer (Böckelman et al., 2011), gastric cancer (Khanna et al., 2009), non-small-cell lung cancer (Dong et al., 2011), and head and neck squamous cell carcinoma (HNSCC) (Böckelman et al., 2011). It is also overexpressed in testicular stem cells to regulate the proliferation of spermatogonial cells; in the spermatogonial cells of CIP2A mutant mice, promyelocytic leukemia zinc finger and other stem cell renewal-related genes exhibit lower expression, suggesting that CIP2A influences testicular stem cells and progenitor cells (Ventelä et al., 2015). In tongue cancer, CIP2A expression is positively correlated with tongue hyperplasia, which displays strong local inflammation (Seppälä et al., 2015). CIP2A is overexpressed in the fibroblast-like synoviocytes of rheumatoid arthritis cases, producing high levels of inflammatory cytokines (Lee et al., 2012). However, the relationship between CIP2A and testicular inflammation is unclear.

As demonstrated in a previous study, CIP2A is a target gene of OCT4 that is involved in HNSCC and radio-resistance (Ventelä et al., 2015). This study was conducted to ascertain whether OCT4 and CIP2A play important roles in inflammation, cell death, and redox equilibrium during orchitis. OCT4 and CIP2A levels were compared among non-induced Sertoli (supporting), Leydig (interstitial), and germ (reproductive) cells, as well as in these testicular cell types after stimulation with lipopolysaccharide (LPS). In addition, the roles of OCT4 and CIP2A in mediating LPS-induced testicular cell abnormalities were elucidated.

## Materials and Methods

### Animals

Male C57BL/6 mice (*n* = 16) were purchased from the Experimental Animal Center (PUMC, Beijing, China) and maintained in pathogen-free conditions with controlled temperature and humidity, light (12/12 h light/dark), and free access to food and water. Animal experiments met the guidelines for the Care and Use of Laboratory Animals approved by the Chinese Committee of Animal Care. This study was approved by the Second Affiliated Hospital and Yuying Children’s Hospital of Wenzhou Medical University.

### LPS Injection in the Testes

First, 50 mg/kg of sodium pentobarbital was injected to anesthetize the mice prior to surgical exposure of their testes. In the experimental group of mice (*n* = 8), each testis was injected with a solution comprising 0.3 μg of LPS in 10 μL of phosphate-buffered saline (PBS). In the control group (*n* = 8), 10 μL of PBS was injected into each testis. The mRNA and protein levels of several orchitis-associated factors in the testes were evaluated 12 h after LPS injection.

### Immunohistochemistry

Rabbit polyclonal antibody anti-IL-1β that recognizes mouse IL-1β (GeneTex, Irvine, CA, United States) was used to detect the expression and localization of IL-1β in acetone-fixed frozen testis sections (7 μm thick). Sections were incubated with 5% normal goat serum and 0.03% Triton X–100 containing 4% bovine serum albumin for IL-1β for 30 min at room temperature, and treated with avidin/biotin blocking solution (Vector Lab., Burlingame, CA, United States) followed by overnight incubation with the primary antibody IL-1β (1/500) at 4°C in a humidified chamber. A biotinylated goat anti-rabbit IgG (1/250, Vector Lab.) was used as the secondary antibody. Endogenous peroxidase activity was blocked by treatment with 0.01% H_2_O_2_ in methanol for 30 min. The reaction was amplified with the Vectastain Elite ABC Kit (Vector Lab.), and the reaction product was visualized by the addition of diaminobenzidine substrate (Vector Lab.). Sections were counterstained with hematoxylin. Negative controls were obtained by incubating sections with PBS instead of primary antibodies.

### Isolation and Preparation of the Various Testicular Cells

Three-week-old male C57BL/6 mice were used to isolate interstitial and supporting cells, whereas 5-week-old male C57BL/6 mice were used to isolate reproductive cells, according to previously described methods (Shang et al., 2011; Zhu et al., 2013). In brief, the testes from three mice that underwent decapsulation were incubated with collagenase type I (0.5 mg/mL; Sigma, St. Louis, MO, United States) in Dulbecco’s modified Eagle’s medium/nutrient mixture F-12 (DMEM/F12; Life Technologies, Carlsbad, CA, United States) for 15 min with mild oscillation at ambient temperature. The suspension was then filtered through a 80-μm-thick copper mesh to isolate the Leydig cells in spermatogenic tubules. Leydig cells were cultured with DMEM/F12 containing 100 U/mL of penicillin, 10% fetal calf serum (Life Technologies), and 100 mg/mL of streptomycin for 24 h. Thereafter, 0.125% trypsin was employed to isolate the interstitial cells for 5 min; this process did not separate the testicular macrophages. Cell staining with 3-β-hydroxysteroid dehydrogenase (an interstitial cell marker) (Chen et al., 2017) indicated that the interstitial cell purity reached 92%. Immunostaining with macrophage marker F4/80 revealed that the macrophage ratio in the interstitial cell preparation was less than 5%. Other minor cell contaminants might have been fibroblasts and vascular endothelial cells.

Peritubular myoid cells were removed by incubating the seminiferous tubules with collagenase type I at ambient temperature for 15 min. After cutting the microtubules into 1 mm pieces, they were incubated with hyaluronidase (0.5 mg/mL, Sigma) at ambient temperature for 10 min, following which the reproductive cells were separated from the supporting cells through mild pipetting. The suspension was then incubated in DMEM/F12 for 6 h at 32°C, after which the reproductive cells were retrieved by collecting the non-adherent cells. The cell nuclear morphology was elucidated with 4′,6-diamidino-2-phenylindole staining, which revealed that reproductive cells exceeded 95% purity. After 24 h of incubation, the supporting cells were stimulated for 1 min in hypotonic solution containing 20 mM Tris-buffered saline (TBS, pH 7.4) to dislodge any adhered reproductive cells. Immunostaining for the WT1 marker (Eozenou et al., 2020) indicated that the supporting cells presented more than 95% purity.

### Transfection and Treatment of the Testicular Cells

To stimulate cells with LPS, the isolated supporting, interstitial, and reproductive cells were cultured in 12-well plates at 2 × 10^5^ cells per well for 12 h and then induced for 12 h with 2 μg/mL LPS.

To stimulate cells with TD-19, the isolated cells were cultured in 12-well plates at 2 × 10^5^ cells per well for 12 h and then treated for 12 h with 1 μM TD-19.

To overexpress SHP-1 in cells, the isolated cells were grown in six-well plates (2 × 10^5^ cells per well) for 12 h and transfected with 100 nM pcDNA3-OCT4 or pcDNA3-NC (normal control) using the method described above. Following culture for 24 h, the cells underwent treatment with LPS/TD-19 as described above.

### RT-qPCR

Total RNA was extracted from cells using TRIzol reagent according to the manufacturer’s protocol. The extracted RNA was then incubated with RNase-free DNase 1 (Invitrogen, Carlsbad, CA, United States) to remove DNA contaminants. The GAPDH gene was amplified by a 30-cycle polymerase chain reaction (PCR) to verify the deletion of genomic DNA before the reverse transcription reaction. RNA (2 μg) was reverse-transcribed to cDNA in a reaction mixture of 20 μL containing 2 μM dNTPs, 2.5 μM random hexamers, and 200 U M-MLV Reverse Transcriptase (Promega, Madison, WI, United States). A Power SYBR Green PCR Master Mix Kit (Applied Biosystems, Foster City, CA, United States) was employed for PCR on the Applied Biosystems ABI PRISM 7300 real-time cycler. This was followed by a 3 min initial denaturation at 95°C and then 40 cycles at 95°C for 10 s, 58°C for 30 s, and 72°C for 30 s. GAPDH was used as the internal reference. The relative mRNA levels of target genes were determined by the 2^−ΔΔ*CT*^ assay described in the No. 2 User Bulletin of Applied Biosystems (P/N 4303859) (Livak and Schmittgen, 2001).

### Western Blot (Wb)

For the WB analysis of target proteins, testes or isolated cells were lysed with lysis buffer, and protein concentrations were determined using BCA protein assay kits (Pierce Biotechnology, Rockford, IL, United States). Next, each protein sample (15 μg) was separated by 10% sodium dodecyl sulfate-polyacrylamide gel electrophoresis, and the protein bands were electrotransferred to a polyvinylidene fluoride (PVDF) membrane (Millipore, Burlington, MA, United States). After blocking for 1 h with 5% skim milk powder in TBS (pH 7.4), the membrane was incubated with the primary antibodies overnight at 4°C. Following two rinses with 0.1% TBS-Tween, the PVDF membrane was incubated with horseradish peroxidase-conjugated secondary antibodies for 1 h at ambient temperature. The antigen–antibody complexes were visualized with ECL detection kits (Zhongshan Biotechnology, Zhongshan, China), with β-actin serving as the loading control.

### Enzyme-Linked Immunosorbent Assay (ELISA)

At 12 h after stimulation of the isolated cells with LPS, the supernatant medium was obtained from the cell cultures to detect secreted interleukin-1β (IL-1β), IL-6, tumor necrosis factor-α (TNF-α), monocyte chemoattractant protein-1 (MCP-1), interferon-α (IFN-α), and IFN-β proteins with ELISA kits (R&D Systems, Minneapolis, MN, United States). Absorbance was detected at 405 nm with a Bio-Rad Laboratories Model 680 microplate reader (Bio-Rad, Hercules, CA, United States). The concentration of each protein was then determined from its standard curve according to a previously described method (Nahid et al., 2009).

### Flow Cytometry

Flow cytometry was performed to quantify the dead cell count using Annexin V-FITC/PI apoptosis detection kits (BD Pharmingen, San Diego, CA, United States). In brief, the cell suspension was incubated with 10 μL of Annexin V-FITC and 5 μL of propidium iodide following preparation with 20 μL of binding buffer. The apoptotic cells were evaluated and identified using the BD FACSCalibur flow cytometer (BD Biosciences, San Jose, United States) with FACS Diva software.

### Dihydroethidium (DHE) Staining

Cells cultured on 96-well chamber slides were washed with PBS three times for 5 min per wash, and then the slides were incubated with ROS Fluorescent Probe-DHE (Vigorous Biotechnology Beijing Co., Ltd., Beijing, China) in serum-free DMEM F12 medium for 30 min at 37°C in darkness, and fixed in 4% paraformaldehyde for 30 min at room temperature. The cells were washed again and mounted. Immunofluorescence signals were read by SpectraMax M Series Microplate Reader M5.

### Statistical Processing

All data were expressed as the mean ± standard deviation. Differences were evaluated between multiple groups and two groups using analysis of variance and two-tailed Student’s *t*-test and were considered statistically significant when the *P*-value was < 0.05.

## Results

### OCT4 and CIP2A Expression Levels Were Repressed During LPS-Induced Orchitis

**FIGURE 1:**
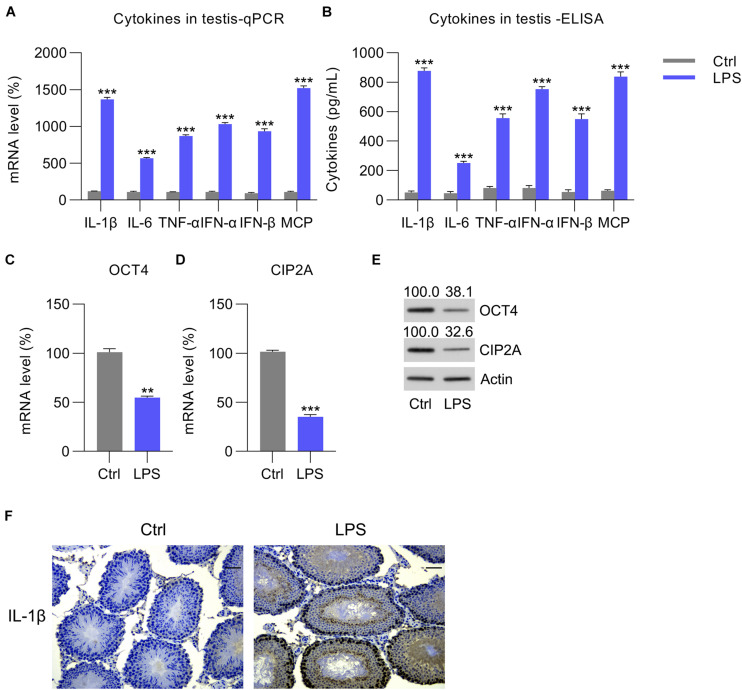
LPS-induced cytokine expression in mouse testes. Overall, 50 mg/kg of pentobarbital sodium was used to anesthetize mice for the subsequent procedure to expose the testes. The testes of the experimental mice were injected with LPS, whereas those of the control animals received PBS. At 12 h post-injection, each testis was homogenized, and the homogenate was tested to determine the **(A)** mRNA and **(B)** protein levels of IL-1β, IL-6, TNF-α, MCP-1, IFN-α, and IFN-β by RT-qPCR and ELISA, respectively. **(C,D)** The mRNA and **(E)** protein levels of OCT4 and CIP2A in the testicular homogenate are shown. **(F)** IHC picture showed IL-1β expression in the testes of Ctrl and LPS group. Scale bar, 100 μm. Data are expressed as the means ± SDs. **P < 0.01, ***P < 0.001, Ctrl vs. LPS.

To study whether OCT4 and CIP2A participated in orchitis, LPS was administered to induce an inflammatory response in mouse testes. We then examined the levels of three pro-inflammatory factors; namely, IL-1β, IL-6, and TNF-α, as well as MCP-1, IFN-α, and IFN-β in the interstitial Leydig, supporting Sertoli, and reproductive germ cells of LPS-treated mouse testes. After LPS injection for 12 h, RT-qPCR and ELISA revealed a robust increase in mRNA and protein expression of the six abovementioned factors in the LPS-treated group compared with that in the control group (Figures 1A,B), suggesting successful establishment of the *in vivo* orchitis model. Next, OCT4 and CIP2A expression in testes was examined. RT-qPCR and WB data revealed that OCT4/CIP2A mRNA and protein levels were decreased following LPS injection compared with those in the control group (Figures 1C–E). Furthermore, IHC staining also confirmed that IL-1β expression was upregulated in the LPS group, compared with control group (Figure 1F).

**FIGURE 2:**
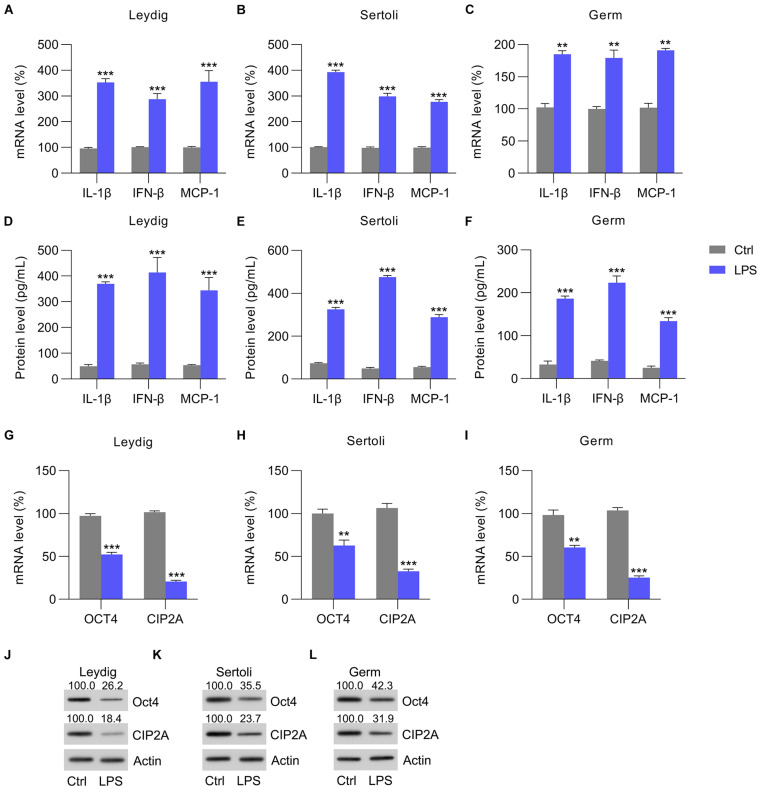
LPS-induced cytokine expression. Sertoli, germ, and Leydig cells isolated from C57BL/6 mice were incubated with LPS or PBS for 12 h. **(A–C)** Total RNA was extracted to determine the relative mRNA expression of IL-1β, IFN-β, and MCP-1 in cells with RT-qPCR. **(D–F)** ELISA was conducted to detect secreted IL-1β, IFN-β, and MCP-1 levels. **(G–I)** The mRNA and **(J–L)** protein levels of OCT4 and CIP2A in testicular homogenate were revealed by RT-qPCR and WB, respectively. Data are expressed as the means ± SDs. **P < 0.01, ***P < 0.001, Ctrl vs. LPS.

To confirm the reduction in OCT4 and CIP2A expression in the testes with inflammation, isolated Leydig, Sertoli, and germ cells were also treated with LPS to induce inflammation. The RT-qPCR data indicated that the mRNA levels of chemokines, pro-inflammatory cytokines, and interferons were increased in the Leydig, Sertoli, and germ cells at 12 h post-LPS stimulation (Figures 2A–C), and these results were supported by the ELISA data (Figures 2D–F). Next, the expression of OCT4 and CIP2A was detected by RT-qPCR and WB in these three cell types. The data showed that OCT4 and CIP2A levels were significantly reduced in Leydig (Figures 2G,J), Sertoli (Figures 2H,K), and germ cells (Figures 2I,L) at 12 h post-LPS treatment compared with those in the untreated control group.

### OCT4 Overexpression Repressed LPS-Stimulated Inflammation in the Isolated Testicular Cells

**FIGURE 3:**
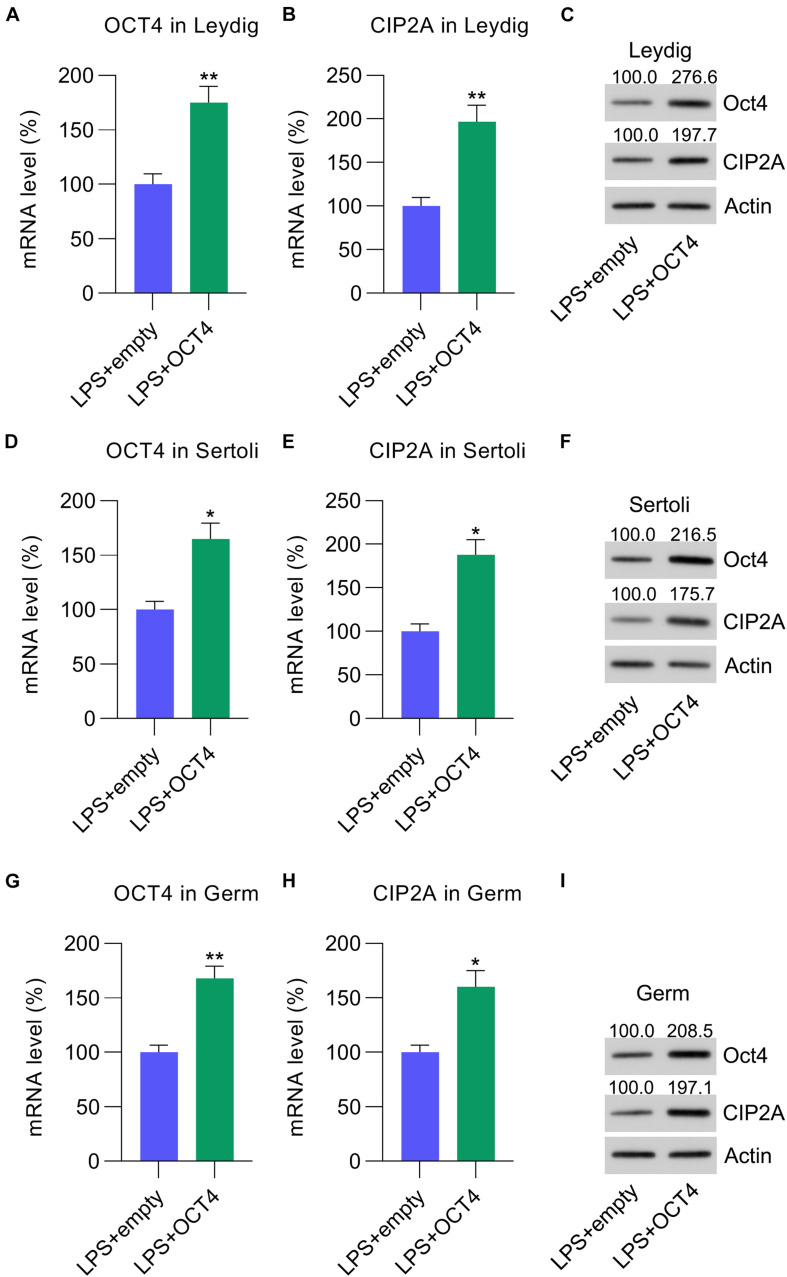
Overexpression of OCT4 in isolated Leydig, Sertoli, and germ cells. Leydig, Sertoli, and germ cells were transfected with pcDNA3-OCT4 or pcDNA3-NC and then incubated with LPS for 12 h. **(A,B,D,E,G,H**) RT-qPCR revealed mRNA levels and **(C,F,I)** WB showed the protein levels of OCT4 and CIP2A in the cells. Data are expressed as the means ± SDs. *P < 0.05, **P < 0.01, LPS + empty vs. LPS + OCT4.

To evaluate the effect of the OCT4-CIP2A axis in LPS-induced testicular inflammation, Leydig, Sertoli, and germ cells were transfected with pcDNA3-OCT4 to overexpress OCT4, while cells transfected with pcDNA3-NC served as controls. RT-qPCR and WB data showed that OCT4 expression was upregulated after pcDNA3-OCT4 transfection in Leydig (Figures 3A,C), Sertoli (Figures 3D,F), and germ cells (Figures 3G,I). A previous study indicated that OCT4 is positively correlated with CIP2A expression (Ventelä et al., 2015). As expected, following OCT4 overexpression, the CIP2A mRNA and protein levels were remarkably increased in Leydig (Figures 3B,C), Sertoli (Figures 3E,F), and germ cells (Figures 3H,I).

**FIGURE 4:**
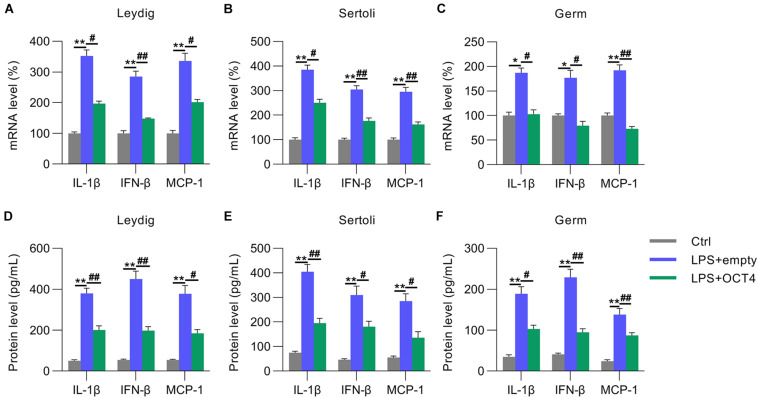
Role of OCT4 overexpression in inflammation. Leydig, Sertoli, and germ cells were transfected with pcDNA3-OCT4 or pcDNA3-NC and then incubated with LPS for 12 h. **(A–C)** Total RNA was extracted, and the relative mRNA levels of IL-1β, IFN-β, and MCP-1 in the cells were determined using RT-qPCR. **(D–F)** ELISA was conducted to examine secreted IL-1β, IFN-β, and MCP-1 levels in the cells. Data are expressed as the means ± SDs. *P < 0.05, **P < 0.01, Ctrl vs. LPS + empty; ^#^*P* < 0.05, ^##^*P* < 0.01, LPS + empty vs. LPS + OCT4.

We then detected the expression of IL-1β, MCP-1, and IFN-β in LPS-treated testicular cells with or without OCT4 overexpression. RT-qPCR data indicated that mRNA levels were significantly repressed in Leydig, Sertoli, and germ cells after OCT4 upregulation compared with those in the control group (Figures 4A–C). The ELISA data from the supernatant medium supported these results (Figures 4D–F).

### TD-19 Administration Counteracted the Effect of OCT4 Overexpression on LPS-Induced Inflammation in Isolated Testicular Cells

**FIGURE 5:**
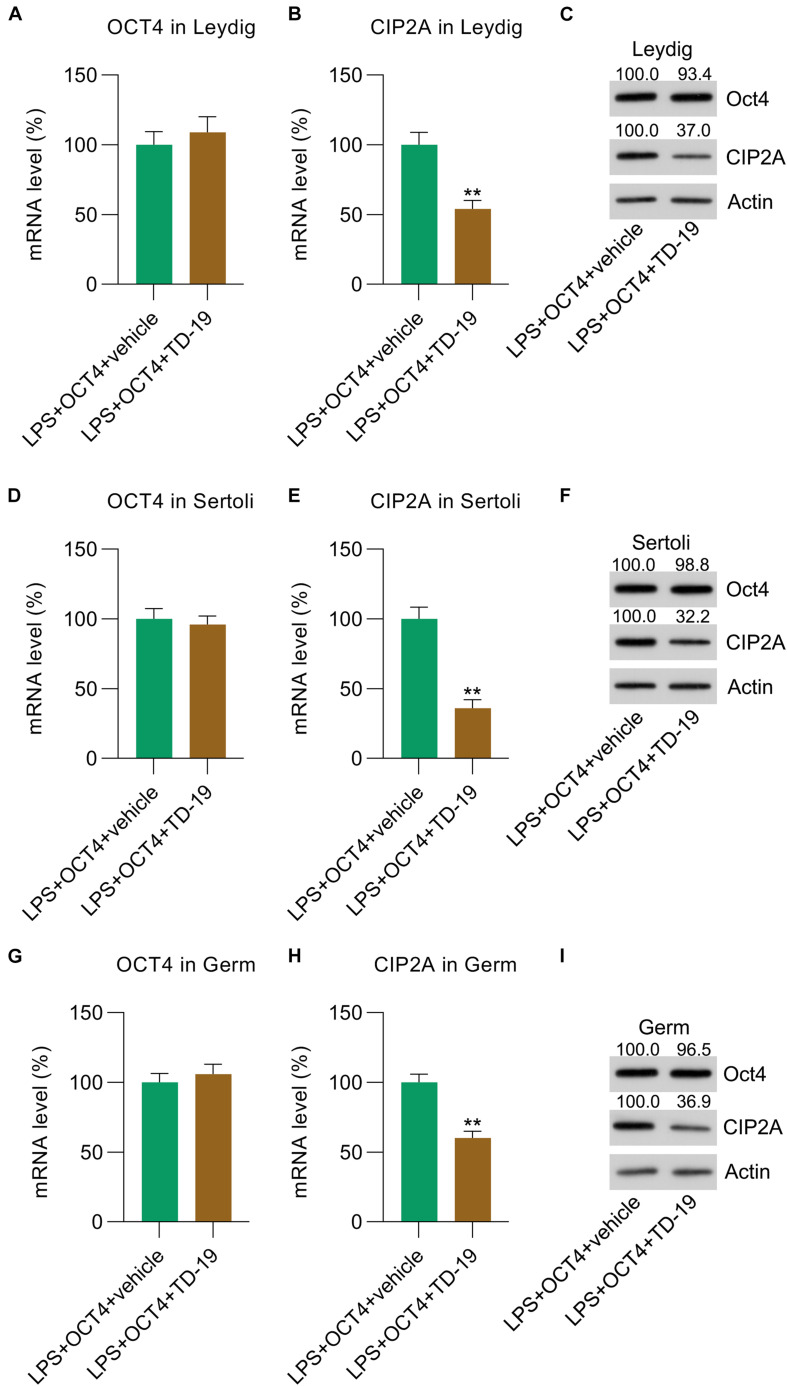
TD-19 administration in OCT4-overexpressing and LPS-induced Sertoli, Leydig, and germ cells. Leydig, Sertoli, and germ cells were transfected with pcDNA3-OCT4 or pcDNA3-NC and then incubated with LPS and TD-19 for 12 h. **(A,B,D,E,G,H)** RT-qPCR showed the mRNA expression and **(C,F,I)** WB revealed the protein expression of OCT4 and CIP2A in the cells. Data are expressed as the means ± SDs. **P < 0.01, LPS + OCT4 + vehicle vs. LPS + OCT4 + TD-19.

To probe the involvement of CIP2A in LPS-induced inflammation inhibited by OCT4 in testicular cells, OCT4-overexpressing LPS-treated Leydig, Sertoli, and germ cells were treated with TD-19, a CIP2A inhibitor (Chao et al., 2014). RT-qPCR results indicated that treatment with TD-19 did not cause any alteration in OCT4 expression at the mRNA level in the three cell types compared with that in the vehicle-treated group (Figures 5A,D,G), while mRNA expression of CIP2A was significantly downregulated after TD-19 administration (Figures 5B,E,H). In addition, the WB data confirmed that the CIP2A protein level was reduced by TD-19 treatment; however, OCT4 expression did not change between the TD-19- and vehicle-treated groups (Figures 5C,F,I).

**FIGURE 6:**
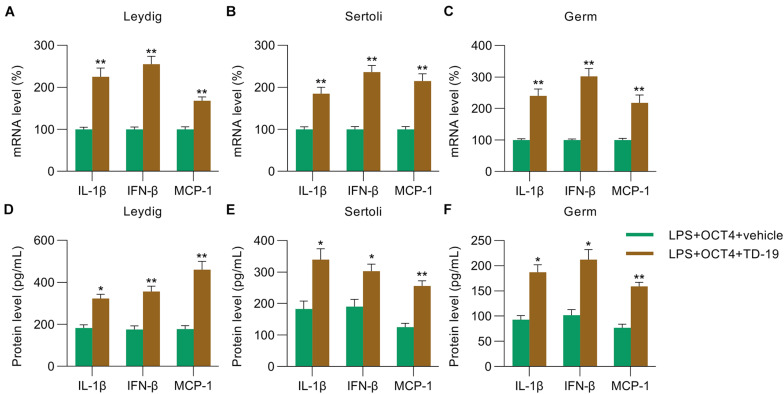
Effects of TD-19 administration on inflammation in OCT4-overexpressing and LPS-induced Sertoli, Leydig, and germ cells. Leydig, Sertoli, and germ cells were transfected with pcDNA3-OCT4 or pcDNA3-NC and then incubated with LPS and TD-19 for 12 h. **(A–C)** Total RNA was extracted from the cells, and the relative mRNA levels of IL-1β, IFN-β, and MCP-1 in the cells were determined using RT-qPCR. **(D–F)** ELISA was conducted to examine the secreted IL-1β, IFN-β, and MCP-1 levels in the cells. Data are expressed as the means ± SDs. *P < 0.05, **P < 0.01, LPS + OCT4 + vehicle vs. LPS + OCT4 + TD-19.

Next, RT-qPCR and ELISA were utilized to monitor the IL-1β/IFN-β/MCP-1 levels in Leydig, Sertoli, and germ cells with or without TD-19 administration. RT-qPCR and ELISA data indicated that these expression levels were promoted in the three testicular cell types in the TD-19 group compared with those in the vehicle-treated group (Figures 6A–F).

### The OCT4-CIP2A Axis Was Involved in Inflammation-Associated Apoptotic Death in LPS-Treated Testicular Cells

**FIGURE 7:**
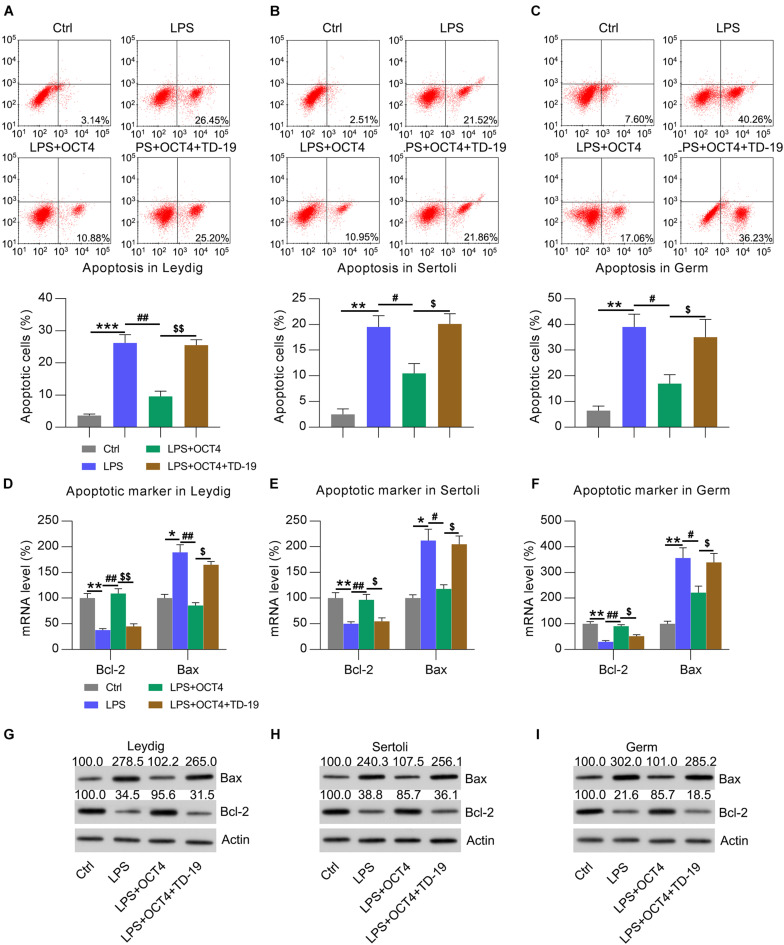
Effects of OCT4 and CIP2A on apoptosis of LPS-induced Sertoli, Leydig, and germ cells. Leydig, Sertoli, and germ cells were transfected with pcDNA3-OCT4 or pcDNA3-NC and then incubated with LPS and TD-19 for 12 h. **(A–C)** The apoptotic cell count was evaluated using Annexin V-FITC/PI flow cytometry and is displayed in the right quadrant in each plot. The apoptotic rates of each cell type are presented in the lower panel. **(D–F)** RT-qPCR detection of the mRNA levels and **(G–I)** WB analysis of the protein expression of Bcl-2 and Bax in the cells. Data are expressed as the means ± SDs. *P < 0.05, **P < 0.01, ***P < 0.001, Ctrl vs. LPS; ^#^*P* < 0.05, ^##^*P* < 0.01, LPS vs. LPS + OCT4; ^$^*P* < 0.05, ^$$^*P* < 0.01, LPS + OCT4 vs. LPS + OCT4 + TD-19.

Considering that orchitis is always accompanied by testicular injury, we utilized flow cytometry and apoptotic marker (Bcl-2 and Bax) analysis to quantify the numbers of apoptotic Leydig, Sertoli, and germ cells following stimulation with LPS for 12 h. Compared with that in untreated control cells, there was an obvious increase in apoptosis among these cells after LPS administration (Figures 7A–C). In OCT4-overexpressing LPS-treated cells, the apoptotic cell number was obviously reduced; however, TD-19 treatment contributed to restoring the apoptotic cell proportions of Leydig, Sertoli, and germ cells (Figures 7A–C). To confirm these findings, we detected the mRNA and protein levels of Bax and Bcl-2 in the three cell types with various treatments (Figures 7A–C). The findings revealed that Bcl-2 expression was notably decreased whereas Bax expression was elevated due to LPS stimulation compared to that in untreated cells, while OCT4 overexpression reversed these alterations. In addition, TD-19 treatment in OCT4-overexpressed and LPS-stimulated cells recovered the Bax levels and eliminated Bcl-2 in Leydig, Sertoli, and germ cells (Figures 7D–I). These data suggested that the OCT4-CIP2A axis was involved in inflammation-associated apoptotic death in LPS-treated testicular cells.

### The OCT4-CIP2A Axis Played a Role in Inflammation-Related Oxidant Response in LPS-Treated Testicular Cells by Mediating the Keap1-Nrf2-HO-1 Pathway

**FIGURE 8:**
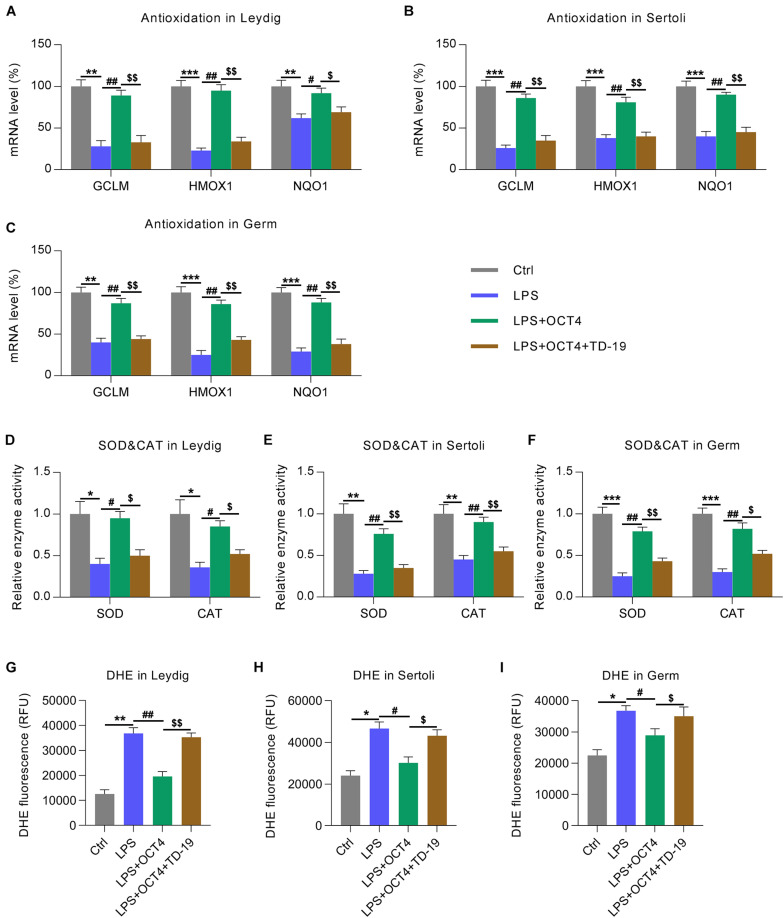
Effects of OCT4 and CIP2A on antioxidant gene expression in LPS-induced Sertoli, Leydig, and germ cells. Leydig, Sertoli, and germ cells were transfected with pcDNA3-OCT4 or pcDNA3-NC and then incubated with LPS and TD-19 for 12 h. **(A–C)** The levels of antioxidation-associated genes in the cells were determined by RT-qPCR. **(D–F)** SOD and CAT activity in the cells was examined. **(G–I)** The DHE assay was carried out to detect the ROS generation in the cells. Data are expressed as the means ± SDs. *P < 0.05, **P < 0.01, ***P < 0.001, Ctrl vs. LPS; ^#^*P* < 0.05, ^##^*P* < 0.01, LPS vs. LPS + OCT4; ^$^*P* < 0.05, ^$$^*P* < 0.01, LPS + OCT4 vs. LPS + OCT4 + TD-19.

Because the reduced antioxidant response was a manifestation of testicular inflammation (Huang et al., 2020), we next determined the expression of antioxidant genes in testicular cells with various treatments using RT-qPCR. Data showed that the expression of antioxidant proteins, including GCLM, HMOX-1, and NQO1 (Wang et al., 2019), was downregulated in the LPS-treated Leydig, Sertoli, and germ cells compared with that in the control group. Furthermore, OCT4 overexpression counteracted the effect of LPS on these levels, while TD-19 administration attenuated antioxidant gene expression in cells (Figures 8A–C). In addition, the LPS-induced downregulation of superoxide dismutase (SOD) and catalase (CAT) activities were restored in the three cell types upon OCT4 overexpression, whereas the restored SOD and CAT activities were further impaired by TD-19 (Figures 8D–F). DHE assay was performed to detect the ROS level in the control, LPS, LPS + OCT4, and LPS + OCT4 + TD-19 conditions. A higher ROS level was found in the LPS-induced testicular cells compared to in the control group, while OCT4 overexpression partially ameliorated the production of ROS. However, TD-19 administration counteracted the effect of OCT4 overexpression on ROS generation (Figures 8G–I). To investigate whether the redox response was downstream effectors that contribute to inflammation-induced cell apoptotic death, pro-oxidants (paraquat) and anti-oxidants (NAC) were also utilized to administrate the LPS-induced testicular cells. The results showed that Paraquat treatment augmented ROS generation (Supplementary Figures 1A–C) and cell apoptosis (Supplementary Figures 1D–F), which was triggered by LPS, while NAC administration led to reduced ROS levels and ameliorated apoptosis (Supplementary Figures 1A–F).

**FIGURE 9:**
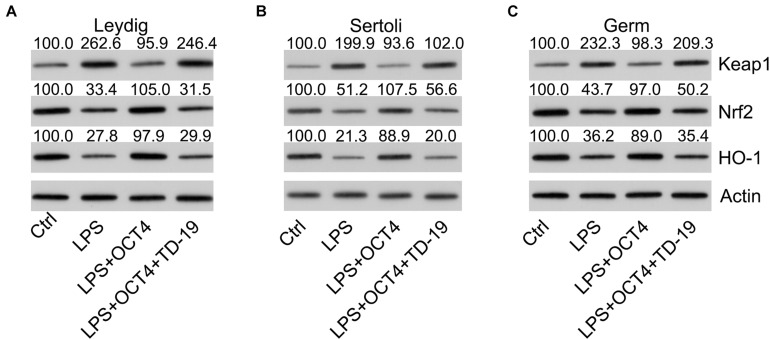
Effects of OCT4 and CIP2A on the activation of the Keap1-Nrf2-HO-1 pathway in LPS-induced Sertoli, Leydig, and germ cells. Leydig, Sertoli, and germ cells were transfected with pcDNA3-OCT4 or pcDNA3-NC and then incubated with LPS and TD-19 for 12 h. **(A–C)** The levels of Keap1, Nrf2, and HO-1 in the cells were determined by WB.

To assess whether the Keap1-Nrf2-HO-1 axis, a well-recognized antioxidant pathway, participated in OCT4-CIP2A-mediated LPS-induced orchitis in testicular cells, we detected the protein expression of Keap1, HO-1, and Nrf2 using WB. According to the WB data, Keap1 expression in the three testicular cell types increased and Nrf2 and HO-1 expression decreased by LPS stimulation, while OCT4 upregulation reversed these effects. After TD-19 administration in OCT4-overexpressing and LPS-induced Leydig, Sertoli, and germ cells, Keap1 expression was upregulated and Nrf2 and HO-1 expression was downregulated, suggesting that the OCT4-CIP2A axis played a role in the inflammation-related oxidant response in LPS-treated testicular cells by mediating the Keap1-Nrf2-HO-1 pathway (Figures 9A–C).

## Discussion

Both OCT4 and CIP2A, which are highly expressed in human cancers, are associated with low survival and poor resistance after patients receive DNA-damaging treatments (Tsai et al., 2011; Shen et al., 2014). Meanwhile, OCT4 and CIP2A have been positively associated with inflammation in many diseases (Yasuda et al., 2011; Lee et al., 2012; Seppälä et al., 2015; Li et al., 2017). However, few studies have examined their functions in pathologies related to orchitis. In this study, OCT4 and CIP2A were found to play regulatory roles in the inflammation, apoptosis, and redox disorder of Leydig, Sertoli, and germ cells after LPS stimulation.

Orchitis makes testes susceptible to various pathogens present in the blood or urogenital tract. Testicular cells overcome the immune privilege and produce a suitable and effective local response to invasive pathogens. Such antimicrobial responses are a result of the organ’s natural immunity. The innate immunity of testes in orchitis is important when the immune function of the body is damaged. The function of OCT4 in the radio-resistance of multiple cancers is linked to its role in maintaining the resistance to DNA damage in cancer stem cells, and thus, the regeneration of progenitor cells. In a mouse model of LPS-induced acute uterine injury, which was featured by acute inflammation (Weiss et al., 2009), there was a significant upregulation of OCT4 at 12 h, compared with the values before LPS or PBS injection (Xiao et al., 2017). In inflamed ulcerative colitis-associated colorectal cancer, the expression of OCT4 increased. Li et al. (2017) demonstrated that OCT4 upregulation in human adipose tissue-derived mesenchymal stem cells reduced the expression of pro-inflammatory cytokines, suggesting that OCT4 possessed an anti-inflammatory effect. However, knowledge regarding OCT4-targeted genes that are involved in apoptosis, inflammation, and antioxidant response remains limited. Here, OCT4 expression was found to be downregulated in LPS-treated testes and LPS-stimulated isolated testicular cells (Leydig, Sertoli, and germ cells). It was also found that LPS could induce an inflammatory response, apoptotic death, and antioxidant response in testicular cells, accompanied by the reduced expression of OCT4. These inflammatory and apoptotic phenomena were repressed by OCT4 overexpression, indicating that OCT4 exerts an inhibitory effect on testicular inflammation and injury, which is consistent with the findings of a previous study (Li et al., 2017).

A previous study concluded that CIP2A is a novel target of OCT4; thus, CIP2A induces cellular radio-resistance owing to its regulatory effects on cell stemness (Ventelä et al., 2015). CIP2A-mediated regulation of the serine/threonine phosphatase activity of PP2A stimulated Akt kinase activity, E2F1 phosphorylation, and some other carcinogenic mechanisms (Shen et al., 2014; Wang et al., 2019). Shentu et al. (2019) demonstrated that CIP2A upregulation in astrocytes induces astrogliosis and the release of cytokines and Aβ in Alzheimer’s disease, indicating that CIP2A plays a pro-inflammatory role in Alzheimer’s disease. Our results showed that the loss of OCT4 expression resulted in a reduction of the CIP2A levels in LPS-stimulated testes and testicular cells, which is accompanied by robust inflammation and apoptosis. Intriguingly, inhibition of CIP2A by TD-19 administration abolished the effect of OCT4 overexpression on inflammation, apoptosis, and antioxidant response in LPS-treated testicular cells, suggesting an anti-inflammation effect of CIP2A in orchitis. Altogether, these results highlighted the association between OCT4 and CIP2A in LPS-induced testicular cells during orchitis.

Oxidative stress is defined as an imbalance between the production of free radicals and reactive metabolites or ROS. This imbalance leads to the inflammation and injury of cells (Tu et al., 2019). To maintain oxidant homeostasis in cells, many regulators exert remarkable effects on intracellular antioxidant defenses. Nrf2 binds to enhancer sequences termed androgen response elements to act as a key regulator of the expression of GST, NQO1, and other antioxidant enzymes (Zhang, 2010; Ma and He, 2012). The expression and activity of Nrf2 are tightly repressed through its binding to the Keap1 ligase complex in the cytoplasm; thus, Nrf2 translocation from the cytosol to the nucleus is limited. Consequently, constitutive Nrf2 levels act as a regulator of the basal antioxidant levels (Ma and He, 2012). Keap1-Nrf2 pathway activation can promote the release of some cytokines, neurotic apoptotic death, and generation of superoxide products (Satoh et al., 2006, 2009; Li et al., 2018). OCT4 has been postulated to be involved in enhancing pro-survival pathways and antioxidant defenses. Gupta and Storey (2020) observed an increment of OCT4 expression after 24 h of anoxia and 4 h of reoxygenation in the liver and skeletal muscle of wood frogs. Meanwhile, several studies suggested that oxidative stress increases the activity of PP2A, downstream of CIP2A (Elgenaidi and Spiers, 2019). However, direct evidence for how OCT4 and CIP2A are involved in the cellular antioxidative response is not currently available. Here, we found that OCT4-CIP2A overexpression contributes to the antioxidant response in Leydig, Sertoli, and germ cells, as evidenced by an activated Keap1-Nrf2 axis, reduced ROS production, and upregulated expression of HO-1 and antioxidant genes. Inhibition of CIP2A expression in LPS-treated testicular cells repressed the activation of the Keap1-Nrf2 pathway, increased ROS generation, and inhibited antioxidant levels, thus, confirming the protective effects of OCT4 and CIP2A against oxidative stress in LPS-induced orchitis.

In conclusion, we suggest that OCT4 and CIP2A play a role in improving the function of testes and isolated Leydig, Sertoli, and germ cells after LPS-induced orchitis, whereas OCT4 overexpression contributes to regulating immune reactions in the testes against the simulated viral infection by acting as a positive modulator of CIP2A during orchitis. These findings support the significance of the OCT4–CIP2A axis for congenital immune reactions in testicular inflammation. Taken together, these results warrant the further investigation of OCT4 and CIP2A as two potential therapeutic targets of orchitis.

## Data Availability Statement

The original contributions presented in the study are included in the article/Supplementary Material, further inquiries can be directed to the corresponding author/s.

## Ethics Statement

The animal study was reviewed and approved by the Animal experiments met the guidelines for the Care and Use of Laboratory Animals approved by the Chinese Committee of Animal Care. This study was approved by The Second Affiliated Hospital and Yuying Children’s Hospital of Wenzhou Medical University.

## Author Contributions

RZ, CJ, JZ, and LQ conceived the study and designed the experiments. CZ, SL, and SQ contributed to the data collection. JP and LW performed the data analysis and interpreted the results. RZ and CJ wrote the manuscript. JZ and LQ contributed to the critical revision of article. All authors read and approved the final manuscript.

## Conflict of Interest

The authors declare that the research was conducted in the absence of any commercial or financial relationships that could be construed as a potential conflict of interest.

## Publisher’s Note

All claims expressed in this article are solely those of the authors and do not necessarily represent those of their affiliated organizations, or those of the publisher, the editors and the reviewers. Any product that may be evaluated in this article, or claim that may be made by its manufacturer, is not guaranteed or endorsed by the publisher.

## Supplementary Material

Supplementary Figure 1 Effects of the redox response on cell apoptosis in LPS-induced Sertoli, Leydig, and germ cells. Leydig, Sertoli, and germ cells incubated with LPS and/or paraquat (5 μM)/NAC (50 μM) for 12 h. **(A–C)** The DHE assay was carried out to detect the ROS generation in the cells. **(D–F)** The apoptotic cell count was evaluated using Annexin V-FITC/PI flow cytometry analysis and is displayed in the right quadrant in each plot. The apoptotic rates of each cell type are presented in the lower panel. Data are expressed as the means ± SDs. ^∗^*P* < 0.05, ^∗∗^*P* < 0.01, Ctrl vs. LPS + vehicle; ^#^*P* < 0.05, ^##^*P* < 0.01, LPS + paraquat or NAC vs. LPS + vehicle.
