# Supplementary figures and images for: OCT4 Represses Inflammation and Cell Injury During Orchitis by Regulating CIP2A Expression
Source: Front Cell Dev Biol. 2021 Aug 26;9:683209. doi: 10.3389/fcell.2021.683209 (PMC8427512; doi:10.3389/fcell.2021.683209)

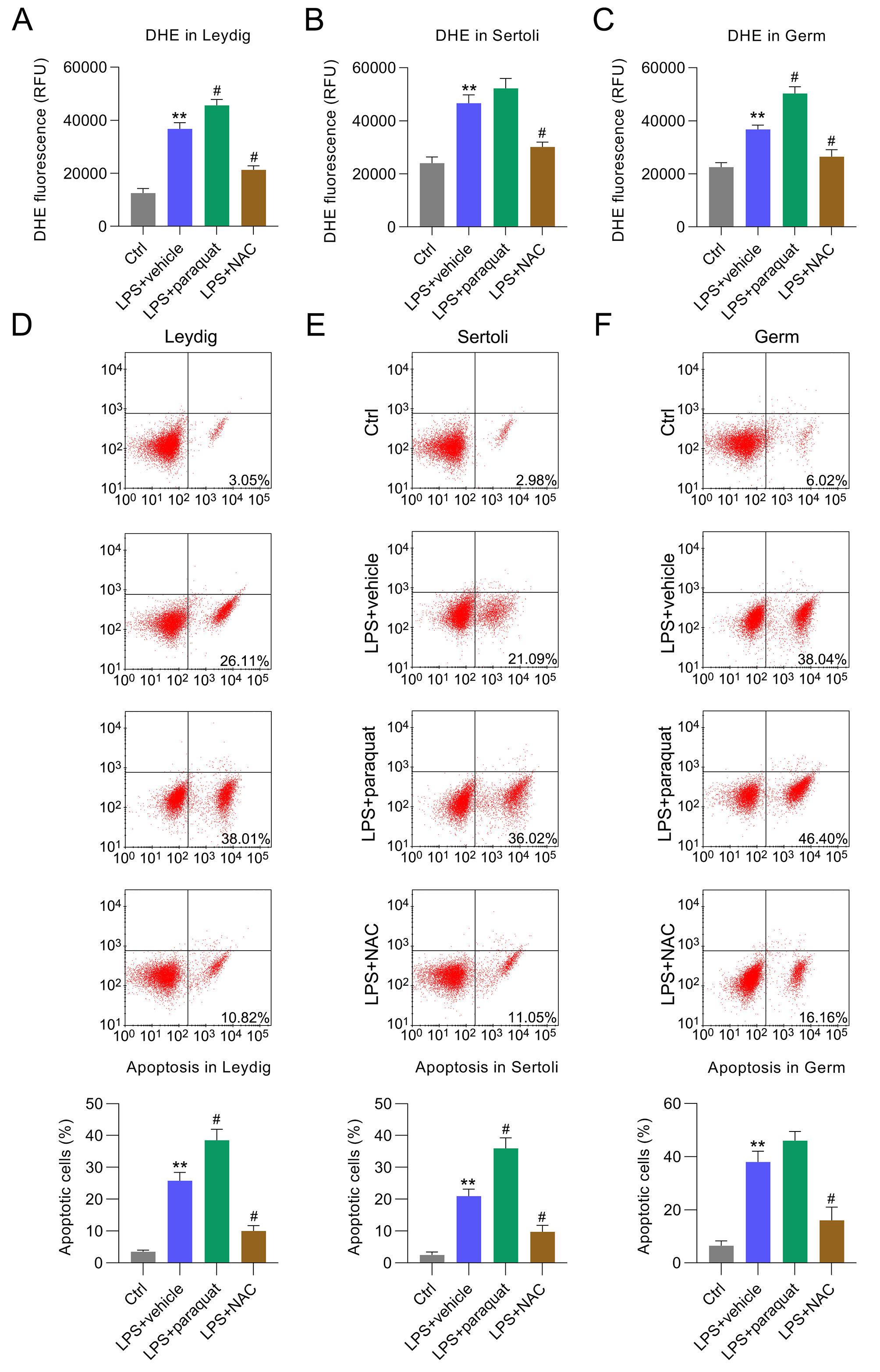

Supplement: Supplementary Figure 1 — Effects of the redox response on cell apoptosis in LPS-induced Sertoli, Leydig, and germ cells. Leydig, Sertoli, and germ cells incubated with LPS and/or paraquat (5 μM)/NAC (50 μM) for 12 h. (A–C) The DHE assay was carried out to detect the ROS generation in the cells. (D–F) The apoptotic cell count was evaluated using Annexin V-FITC/PI flow cytometry analysis and is displayed in the right quadrant in each plot. The apoptotic rates of each cell type are presented in the lower panel. Data are expressed as the means ± SDs. ∗P < 0.05, ∗∗P < 0.01, Ctrl vs. LPS + vehicle; #P < 0.05, ##P < 0.01, LPS + paraquat or NAC vs. LPS + vehicle. [file Image_1.TIF]
